# Collating the knowledge base for the COMET (core outcome measures in effectiveness trials) initiative - a systematic review

**DOI:** 10.1186/1745-6215-14-S1-O67

**Published:** 2013-11-29

**Authors:** Elizabeth Gargon, Binu Gurung, Paula Williamson

**Affiliations:** 1 University of Liverpool, Liverpool, UK

## Background

The COMET Initiative is developing a publically-accessible online resource to collate the knowledge base for core outcome set (COS) development and implementation. This will be used by trial funders, trialists and researchers to see what has been done in their area of interest, and by research funders wishing to fund new work in this area who want to avoid unnecessary duplication of effort. This requires the development and application of an optimal, multi-faceted search strategy to identify work related to the development of COS.

## Objectives

To identify studies that had the aim of determining which outcomes or domains to measure in all clinical trials in a specific condition, and to identify and describe the methodological techniques used in these studies.

## Methods

We developed a multi-faceted search strategy to search electronic databases (MEDLINE via Ovid, SCOPUS and the Cochrane Methodology Register). We contacted Cochrane Review Groups across all areas of health care to request information on COS that they are aware of. We also completed a range of hand searching activities.

## Results

Databases were searched in August 2012 for studies of the selection of outcomes for use in clinical trials. The search identified 24804 potentially relevant abstracts. Screening is ongoing to identify the final set of included studies; it is expected this work will be completed in September 2013. Pilot work suggests that an estimate of up to 1200 relevant papers will be included. Results will be presented.

## Conclusions

This systematic review has identified clinical areas where work has been undertaken, providing the knowledge base for COS development. This review also highlights clinical areas where gaps exist, providing opportunities for future COS development. This is the first step in establishing a database of COS. Ensuring that the database is as comprehensive as possible and keeping it up to date are key to its value for users.

